# Taxonomic studies of the ground beetle subgenus *Falcinebria* Ledoux & Roux, 2005 (Coleoptera, Carabidae, *Nebria*) from Honshû, Japan

**DOI:** 10.3897/zookeys.902.46531

**Published:** 2020-01-13

**Authors:** Kôji Sasakawa

**Affiliations:** 1 Laboratory of Zoology, Department of Science Education, Faculty of Education, Chiba University, 1-33 Yayoi-cho, Inage-ku, Chiba 263-8522, Japan Chiba University Chiba Japan

**Keywords:** Biogeography, cryptic species, endophallus, ground beetle, Japan, male genitalia, phylogeny, taxonomy

## Abstract

Nebria (Falcinebria) reflexa Bates (Carabidae), a Japanese endemic flightless ground beetle, is geographically polytypic and was thought to be composed of four subspecies (including nominotypical subspecies). Populations from Honshû recognized as three subspecies were taxonomically revised based primarily on the shape of the endophallus, a membranous inner sac everted from the aedeagus. Three known taxa, *Nebria reflexa*, *Nebria niohozana* Bates, and *Nebria uenoi* Nakane, are redefined based on endophallus morphology, and the latter two are described as distinct species rather than subspecies of *N. reflexa*. Seven new species are described: *N. sagittata***sp. nov.**, *N. iidesana***sp. nov.**, *N. furcata***sp. nov.**, *N. pisciformis***sp. nov.**, *N. kuragadakensis***sp. nov.**, *N. dichotoma***sp. nov.**, and *N. chugokuensis***sp. nov.** Comparative morphology, primarily of the endophallus, indicated that *N. furcata*, *N. pisciformis*, *N. kuragadakensis*, and *N. uenoi* form basal lineages, and the remaining six species form a clade in which *N. niohozana* and *N. dichotoma* are sister taxa. Species phylogeny and known distributions suggest that the initial diversification of these species occurred in the western Chûbu and eastern Kinki regions of Japan.

## Introduction

Nebria (Falcinebria) reflexa Bates (Carabidae) is a ground beetle first described from Mount Iwaki, Honshû, Japan (Bates 1883). The species occurs within mountainous areas of Honshû, Shikoku, and Kyûshû in the Japanese Archipelago (Uéno 1985) and is geographically polytypic among these areas due to poor dispersal ability caused by atrophied hind wings. Two subspecies on Honshû and one from Kyûshû have been distinguished from the nominotypical subspecies. Among the four known subspecies, *N. r. hikosana* Habu (known from Kyûshû) is readily distinguished based on external morphology (Uéno 1985). Differences among the remaining three, *N. r. reflexa*, *N. r. niohozana* Bates, and *N. r. uenoi* Nakane, are less clear (Mori 2018), and the subspecies identification of populations outside of type localities has been determined largely without evidence (Yoshitake et al. 2011; Yoshimatsu et al. 2018). Thus, a substantial revision of subspecific taxonomy is required (Yoshimatsu et al. 2018).

In some species groups of *Nebria*, the endophallus (a membranous inner sac everted from the aedeagus) is known to be diversified and therefore useful for species-level taxonomy (e.g., Dudko and Shilenkov 2001; Ledoux and Roux 2005; Sasakawa 2016). This genital character had not been examined in *N. reflexa*, but the endophallus is in fact diversified and thus taxonomically useful. This study aims to revise unclear subspecies-level taxonomy for populations of *N. reflexa* on Honshû. To this end, the three subspecies known from Honshû are redefined based on endophallus morphology and, based on these results, taxonomic revisions to other Honshû populations are presented.

## Materials and methods

**Figure 1.**
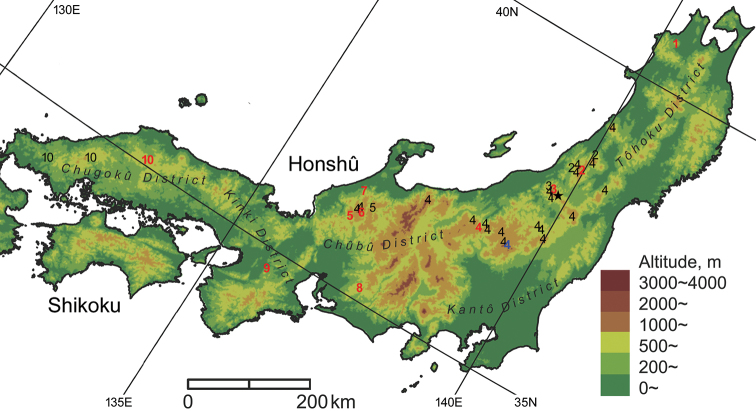
Distribution of 10 species previously classified as *Nebria reflexa* in Honshû. Only records with unambiguous identities (i.e., collection sites of type materials and records based on specimens identified by the endophallus) are presented. **1***N. reflexa* Bates, **2***N. sagittata* sp. nov., **3***N. iidesana* sp. nov., **4***N. niohozana* Bates, **5***N. furcata* sp. nov., **6***N. pisciformis* sp. nov., **7***N. kuragadakensis* sp. nov., **8***N. dichotoma* sp. nov., **9***N. uenoi* Nakane, **10***N. chugokuensis* sp. nov. indicates the locality where the sympatric occurrence of *N. iidesana* and *N. niohozana* was confirmed. Red letters denote the type localities of each species. The blue letter denotes the locality described as the collection site of cotypes of *N. niohozana* but was not designated as the type locality in the lectotype designation.

**Figures 2, 3.**
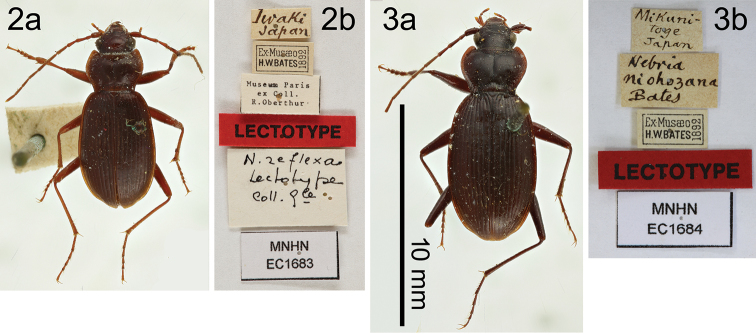
Habitus dorsal views (**a**) and attached labels (**b**) of lectotypes of *Nebria reflexa* (**2**) and *N. niohozana* (**3**).

A total of 120 specimens from various localities in Honshû were examined (Fig. 1). Two specimens, lectotypes of *N. r. reflexa* (♂) and *N. r. niohozana* (♂), which are deposited at the Muséum national d’Histoire naturelle (MNHN), were investigated through high-quality photos that are available for external macromorphological and morphometric studies (Figs 2, 3). In the remaining 118 specimens (72♂, 46♀) examined directly, specimens from the exact type localities of *N. r. reflexa* and *N. r. uenoi* were included, as well as specimens from three sites in the surrounding area (12–14 km) of the type locality of *N. r. niohozana* (Mount Tanigawa, Doai, Mount Naeba). For comparison, specimens from Kyûshû (type materials of *N. r. hikosana*: holotype male and paratype 1♂1♀, deposited at the National Agriculture and Food Research Organization [NARO], Tsukuba, Ibaraki, Japan) were also examined. Examined specimens are preserved in the collections of NARO and in the author’s (KS) collection deposited in the Laboratory of Zoology, Department of Science Education, Faculty of Education, Chiba University, Chiba, Japan.

Body length was measured as the distance from the mandible apices to the end of the elytra. As an index of body shape, the ratio of pronotal posterior margin width (PPW) to elytral length (EL) was determined; PPW was measured as the length between hind angle apices, and EL was measured from the scutellum base to the elytral apex with the elytral lateral margins maintained horizontally. In general, an individual with a smaller PPW/EL has a more slender habitus and more posteriorly constricted pronotum.

To compare genital structures among males, the endophallus was everted by injecting toothpaste at the basal end of the aedeagus. All Honshû specimens except for the lectotypes were dissected. In some older specimens, the gonopore and some surface lobes could not be fully everted, as toothpaste injection can cause the endophallus to burst. To prevent damaging specimens, the endophallus of such specimens was observed with the gonopore protrusion and the lobes not fully everted. Comparison of fully everted gonopore protrusions among species indicated that morphological differences in the protrusion are smaller than those of other lobes. Character observations of incompletely everted lobes on the endophallus surface were complemented by observations of these same lobes everted in specimens collected at, or close to, the same collection site. Terminology of endophallus structures followed Sasakawa (2016), but the new terminology of “dorsomedian lobe” is defined herein as the lobe between the dorsobasal and dorsoapical lobes. To observe female genitalia, muscles around the genitalia were dissolved using 5% potassium hydroxide, and organs were cleaned and observed in pure water.

To confirm species identities based on genital morphology, discriminant analysis based on external morphometrics was performed. In addition to PPW and EL, the following two external body parts were also measured in the lectotypes of *N. reflexa* and *N. niohozana* and 72 male specimens of the 10 species described here: PL, pronotum length along median line; and PAW, pronotal anterior margin width. The linear discriminant analysis was first performed for data set excluding the lectotypes, with “species” as a response variable and the four morphometric measurements (EL, PL, PAW, and PPW) as the explanatory variables. The species identities of the two lectotypes were then determined using the obtained function. All statistical analyses were performed in the statistical package R v.3.4.3 (R Development Core Team 2017). The raw data of the analysis are available in Supplementary material 1, Table S1.

## Taxonomy

**Figure 4.**
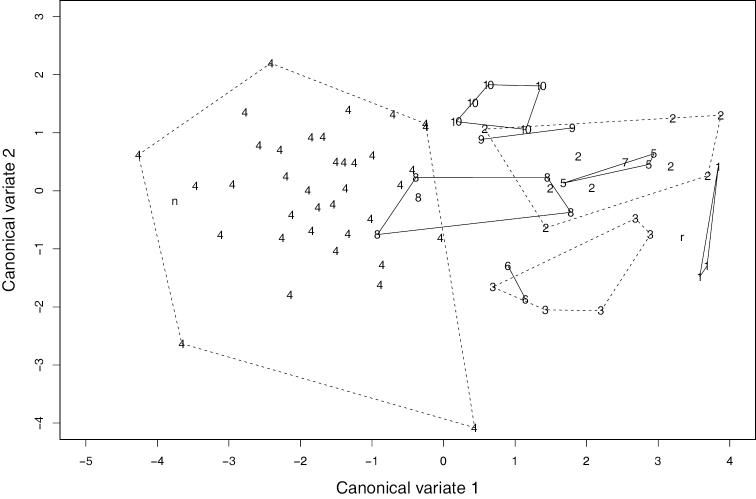
Scatter plot of the first two canonical variates obtained from the discriminant analysis. **1***N. reflexa*, **2***N. sagittata* sp. nov., **3***N. iidesana* sp. nov., **4***N. niohozana*, **5***N. furcata* sp. nov., **6***N. pisciformis* sp. nov., **7***N. kuragadakensis* sp. nov., **8***N. dichotoma* sp. nov., **9***N. uenoi*, **10***N. chugokuensis* sp. nov., **r** lectotype male of *N. reflexa*, **n** lectotype male of *N. niohozana*.

Specimens were classified into 10 species based primarily on the shape of the endophallus. Species identities were further determined by comparing the distribution of each species and the type localities of known taxa (Fig. 1). These results agree with those of the discriminant analysis. In the discriminant function analysis, the lectotypes of *N. reflexa* and *N. niohozana* were classified to *N. reflexa* and *N. niohozana*, respectively, although the former was situated in an intermediate position among *N. reflexa*, *N. iidesana*, and *N. sagittata* on the scatter plot of the first two canonical variates (Fig. 4).

The 10 species described here are similar to one another (Figs 5–16) and share the following adult morphology:

**Figures 5–16.**
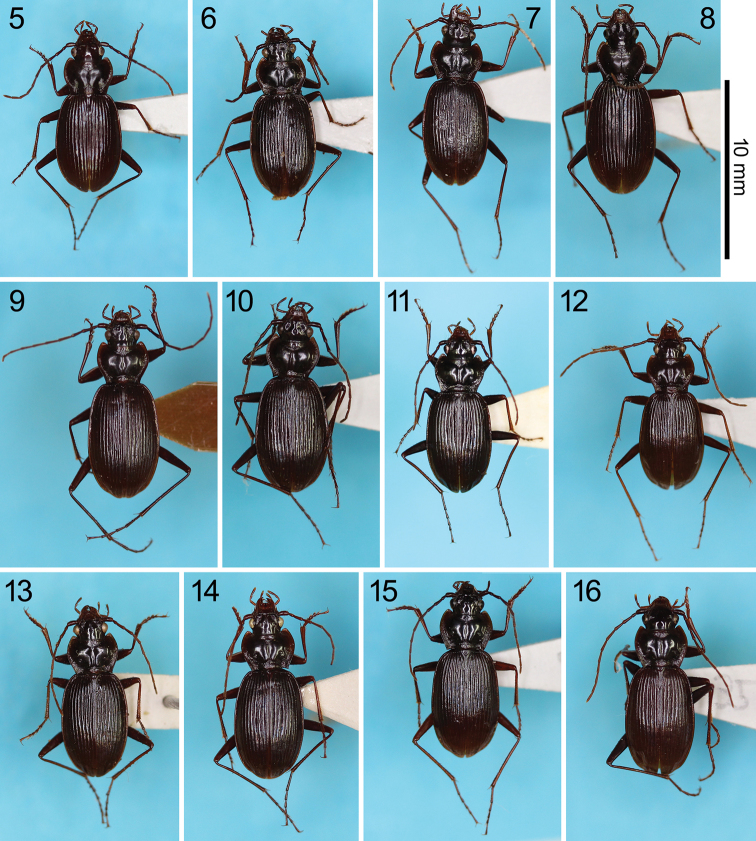
Habitus dorsal view of *Nebria* spp. **5***N. reflexa*, a male from the type locality **6***N. sagittata* sp. nov., holotype male **7***N. iidesana* sp. nov., holotype male **8–10***N. niohozana* males from Mount Chôkai (**8**), Doai, a locality close to the type locality designated for the lectotype (**9**), and Mount Hakusan (**10**); **11***N. furcata* sp. nov., holotype male **12***N. pisciformis* sp. nov., holotype male **13***N. kuragadakensis* sp. nov., holotype male **14***N. dichotoma* sp. nov., holotype male **15***N. uenoi*, a male from the type locality **16***N. chugokuensis* sp. nov., holotype male.

**External characters**: Body flat. Dorsal surface of body reddish black, shiny, not opaque; mouthpart appendages, antennae, legs, pronotal and elytral margins yellowish to reddish brown. Hind wings completely reduced.

Head widest at mid-eye level; eyes large and convex; frontal impression shallow; frons smooth; mentum tooth shallowly bifid. Antennae long, with apices reaching the apical 1/3–1/2 in both sexes.

Pronotum cordate, widest in front of the middle, convex; anterior margin as wide as or slightly wider than posterior margin; lateral margins arcuate in the apical 2/3, straight in the basal 1/3, but more or less sinuate in front of hind angle; anterior angles pronounced, with corners widely rounded; hind angles square to somewhat acute, with sharp corners; lateral margins reflexed throughout; laterobasal impressions large and deep on the posterior part, with the anterior part shallow and reaching the apical half of the pronotum; laterobasal impressions connected by a transverse impression; median line distinct on the central part, but less distinct near the anterior and posterior margins; surface of central part smooth; surface near the posterior margin punctate; surface near lateral and anterior margins punctate in most individuals, but sparser than that of the posterior margin; two marginal setae on each side, anterior setae at widest pronotal point and posterior setae in front of hind angle.

Elytra oblong, widest slightly behind the middle; shoulders and apices rounded, not denticulate; intervals barely convex; scutellar stria present, not connected to stria 1; interval 1 with one setigerous puncture adjoining stria 1 at the level of posterior end of scutellum; interval 3 with five or seven setigerous punctures adjoining or near stria 3.

Ventral surface of the body with punctations on thorax and some sterna, with setae on mentum, gula, and other sterna; meso- and metathorax and sterna 2 and 3 punctate in all individuals, prothorax punctate in some individuals; mentum with two pairs of setae, the anterior pair at the base of mentum tooth and the posterior pair behind the anterior pair; gula with 13–14 setae along the anterior margin; sterna 4–7 with one to three (usually two) setae on each ventrolateral side.

**Male genital characters**: Aedeagus stout and strongly arcuate; apex short and widely rounded. Endophallus with gonopore narrowly protruding; five lobes present on the surface in all species, two on laterobasal surface (laterobasal lobes), two on lateroapical surface (lateroapical lobes), and one on dorsoapical surface (dorsoapical lobe); two additional lobes present in some species, one on the dorsal surface at a position more anterior than the level of the gonopore protrusion (dorsobasal lobe), and the other on the dorsal surface at a position between the level of the gonopore protrusion and dorsoapical lobe (dorsomedian lobe). Both right and left parameres spatulate, with the former larger than the latter.

**Female genital characters**: Gonocoxite 2 semi-triangular and weakly bent posterolaterally. Membranous parts without pigmentation; openings of spermatheca and common oviduct adjacent; innermost part of vagina elongate, forming the bursa copulatrix; spermatheca not uniform in shape between the basal and apical halves; the basal half straight tubular and glued to the wall of the bursa copulatrix; the apical half about half the thickness of the basal half and zigzag tubular.

### Nebria (Falcinebria) reflexa

Taxon classificationAnimaliaColeopteraCarabidae

Bates, 1883

ABFA7326-27C3-5C80-A22D-65D9C19E00B2

**Figures 17–21.**
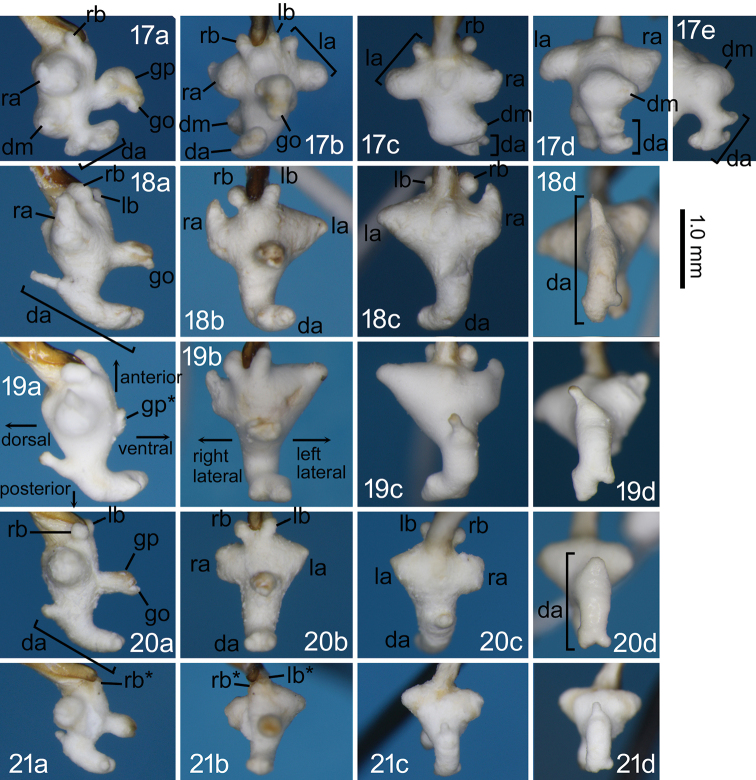
Right lateral (**a**), ventral (**b**), dorsal (**c**), and posterodorsal (**d**) views of the endophallus and the left dorsolateral view of the dorsoapical lobe (**e**) of *Nebria* spp. **17***N. reflexa* male from the type locality **18***N. sagittata* sp. nov., holotype male **19***N. sagittata* sp. nov., a paratype male from “Asahi-mura” **20***N. iidesana* sp. nov., holotype male **21***N. iidesana* sp. nov., paratype male from Mount Kitamata. Abbreviations: da, dorsoapical lobe; dm, dorsomedian lobe; go, gonopore; gp, gonopore protrusion; la, left lateroapical lobe; lb, left laterobasal lobe; ra, right lateroapical lobe; rb, right laterobasal lobe. Asterisk indicates that the gonopore protrusion or lobes are not fully everted.

Figs 2 , 5 , 17 Japanese name: Iwaki-hime-marukubi-gomimushi

Nebria reflexa : Bates (1883): 218 (original description), type locality: “Iwakisan” (originally stated), “Iwaki Japan”, as stated in lectotype designation by Ledoux and Roux (1992: 45); Nakane (1963b): 19 (part); Uéno (1985): 56 (part).
Nebria reflexa reflexa : Ledoux and Roux (1992): 45 (lectotype (♂) designation).
Nebria (Orientonebria) reflexa reflexa : Farkač and Janata (2003): 94.
Nebria (Falcinebria) reflexa reflexa : Ledoux and Roux (2005): 829, plate-fig. 625; Yoshitake et al. (2011): 32; Huber (2017): 50.

#### Notes.

This species is known only from the type locality of Mount Iwaki and has the smallest body size among all species previously regarded as *N. reflexa*. *Nebria reflexa* is distinguished from *N. sagittata* sp. nov. and *N. niohozana*, which are relatively closely distributed and thus may be sympatric, by a T-shaped apex of the dorsoapical lobe. The morphological features of the lectotype male (Fig. 2) are compatible with specimens examined here as *N. reflexa* with respect to all macromorphological features, body length (8.88 mm), and PPW/EL (0.343).

#### Description.

Body length: ♂, 8.92–9.06 mm (mean ± SD: 9.00 ± 0.08 mm, *n* = 3); ♀, 9.19 mm (*n* = 1). PPW/EL: ♂, 0.339–0.356 (mean ± SD: 0.346 ± 0.009, *n* = 3); ♀, 0.312 (*n* = 1). Ventral surface of aedeagal apex not concave. Dorsomedian lobe largely swollen, directed right-laterally. Dorsoapical lobe with the basal part protruding right-dorsolaterally; the protrusion similar in size to the right laterobasal lobe; the apical portion directed right-ventrolaterally, bifurcated in a T shape. Right laterobasal lobe small, with the width from a ventral view narrower than the width of the gonopore protrusion from a lateral view. Left laterobasal lobe small, with the width from a ventral view narrower than the width of the gonopore protrusion from a lateral view. Right lateroapical lobe large, with the apex slightly protruding and bent anteriorly. Left lateroapical lobe bifurcated at the base, with one apex large and directed ventrally and the other similar in size to right laterobasal lobe and directed anteriorly. Ventrobasal surface almost flat, without swelling.

#### Materials examined.

3♂1♀ (NARO), Yunosawa, Mount Iwaki, Aomori Prefecture, Japan, 18.viii.1959, S. Ueno leg.

### Nebria (Falcinebria) sagittata sp. nov.

Taxon classificationAnimaliaColeopteraCarabidae

E0A084D4-D961-545C-85D6-1D8725A2B893

http://zoobank.org/264650A4-C1B0-4347-AB83-5FE19B651D75

Figs 6 , 18 , 19 Japanese name: Asahi-hime-marukubi-gomimushi

Nebria reflexa : Uéno (1985): 56 (part); Nakane (1963b): 19 (part).

#### Notes.

This species is known from the Asahi Mountains and the adjacent Mount Gassan. It is likely sympatric with *N. niohozana* and is distinguished from that species by a smaller body size and a larger PPW/EL. Among known related species, *N. sagittata* sp. nov. is most similar to *N. iidesana* sp. nov., in both external and endophallic structures, but it is distinguished by a strong bend in the apical portion of the right lateroapical lobe.

#### Description.

Body length: ♂, 9.22–9.87 mm (mean ± SD: 9.54 ± 0.26 mm, *n* = 9); ♀, 9.77–10.92 mm (mean ± SD: 10.07 ± 0.43 mm, *n* = 6). PPW/EL: ♂, 0.317–0.345 (mean ± SD: 0.331 ± 0.009, *n* = 9); ♀, 0.307–0.333 (mean ± SD: 0.320 ± 0.009, *n* = 6). Ventral surface of aedeagal apex not concave. Dorsobasal lobe absent. Dorsomedian lobe absent. Dorsoapical lobe with the basal part protruding anterodorsally; the protrusion longer than the right laterobasal lobe; the apical portion directed dorsally, bifurcated at the apex; the left apex more than twice the size of the right apex. Right laterobasal lobe small, with the width from the ventral view narrower than the width of the gonopore protrusion from the lateral view. Left laterobasal lobe small, with the width from the ventral view narrower than the width of the gonopore protrusion from the lateral view. Right lateroapical lobe large, narrowed apically; apical half strongly bent in an anterior direction. Left lateroapical lobe large, narrowed apically. Ventrobasal surface almost flat, without swelling.

#### Type materials.

Holotype: ♂ (KS), Riv. Higuresawa, ca 625 m, Nishikawa-machi, Yamagata Prefecture, Japan (38.320729N, 139.942376E), 9–12.ix.2004, K. Sasakawa leg. Paratypes: 6♂6♀ (KS), same data as the holotype; 1♂ (NARO), “Tachiyazawa-Vill.” [Tachiyazawa, Shônai-machi], Yamagata Prefecture, Japan, 2.viii.1960, Y. Watanabe leg.; 1♂ (NARO), “Asahi-mura” [a part of Murakami-shi], Niigata Prefecture, Japan, K. Baba leg.

#### Etymology.

The specific name derives from the Latin word *sagittata* (arrow-shaped) and refers to the dorsal view of dorsoapical lobe.

### Nebria (Falcinebria) iidesana sp. nov.

Taxon classificationAnimaliaColeopteraCarabidae

94F3CAD0-084A-5AB6-B157-30BC0D7C26E0

http://zoobank.org/1E60020F-32F9-4252-B8D4-CC73E99A1EF1

Figs 7 , 20 , 21 Japanese name: Iide-hime-marukubi-gomimushi

Nebria reflexa : Uéno (1985): 56 (part); Nakane (1963b): 19 (part).
Nebria (Paranebria) reflexa niohozana : Habu and Baba (1972): 3 (part).

#### Notes.

This species is known from the Iide Mountains. Sympatry with *N. niohozana* is considered confirmed based on an observation from the NARO collection, where paper cards affixed to a paratype male of this species and male specimen of *N. niohozana* were held together with the same pin. In external and endophallus characters, *N. sagittata* sp. nov. is a similar species. *Nebria iidesana* is distinguished from *N. niohozana* by a smaller body size and larger PPW/EL, and from *N. sagittata* sp. nov. by a right lateroapical lobe that is not narrowed apically.

#### Description.

Body length: ♂, 9.16–9.87 mm (mean ± SD: 9.53 ± 0.29 mm, *n* = 5); ♀, 10.25–10.64 mm (mean ± SD: 10.39 ± 0.17 mm, *n* = 6). PPW/EL: ♂, 0.319–0.350 (mean ± SD: 0.334 ± 0.013, *n* = 5); ♀, 0.321–0.338 (mean ± SD: 0.330 ± 0.006, *n* = 6). Ventral surface of aedeagal apex not concave. Dorsobasal lobe absent. Dorsomedian lobe absent. Dorsoapical lobe with the basal part protruding anterodorsally; the protrusion slightly smaller than the right laterobasal lobe; the apical portion directed dorsally, bifurcated at the apex; the right and left apices almost similar in size. Right laterobasal lobe small, with the width from a ventral view narrower than the width of the gonopore protrusion from a lateral view. Left laterobasal lobe small, with the width from a ventral view narrower than the width of the gonopore protrusion from a lateral view. Right lateroapical lobe large, almost semispherical, slightly concave at the top. Left lateroapical lobe large, narrowed apically. Ventrobasal surface almost flat, without swelling.

#### Type materials.

Holotype: ♂ (NARO), Mount Takizawamine, alt. 1300 m, Kurokawa, Niigata Prefecture, Japan, 27.vii.1957, K. Baba leg. Paratypes (NARO): 3♂6♀, Kurokawa, Niigata Prefecture, Japan, K. Baba leg. (1♂, Wataba, 25.vii.1957; 1♀, Wataba, 23.vii.1957; 1♀, Wataba, 24.vii.1957; 1♀, Wataba, 25.vii.1957; 1♀, Tamogi, alt. 600 m, 25.vii.1957; 1♂, 18.ix.1959; 1♂2♀, 23.vi.1964); 1♂, Mount Kitamata, the Iide Mountains, Niigata Prefecture, Japan, 15.viii.1964, K. Baba leg.

#### Etymology.

The specific name refers to the Iide Mountains, where the type materials were collected.

### Nebria (Falcinebria) niohozana

Taxon classificationAnimaliaColeopteraCarabidae

Bates, 1883

220BBAB6-53E0-55B0-B433-28DAD1EFEF19

Figs 8–10 , 22–26 Japanese name: Chûbu-hime-marukubi-gomimushi

Nebria reflexa var. Niohozana : Bates (1883): 218 (original description), type locality: “Niohozan” (originally stated), changed to “Mikuni-toge Japan” through lectotype designation by Ledoux and Roux (1992: 42).
Nebria (Paranebria) reflexa niohozana : Habu and Baba (1972): 3 (part).
Nebria reflexa : Uéno (1985): 56 (part).
Nebria reflexa niohozana : Ledoux and Roux (1992): 42, fig. 7b (lectotype (♂) designation); Nakane (1963b): 19, plate 10 fig. 1a (part).
Nebria (Orientonebria) reflexa niohozana : Farkač and Janata (2003): 94.
Nebria (Falcinebria) reflexa niohozana : Ledoux and Roux (2005): 831, plate-fig. 626; Yoshitake et al. (2011): 32 (part); Huber (2017): 50; Yoshimatsu et al. (2018): 37 (part).

#### Notes.

This species was originally described as a variety of *N. reflexa* and was later treated as a subspecies. Although specimens described by Bates (1883) are from “Niohozan” (meaning Mount Nyohô of Nikkô in Tochigi Prefecture), the male specimen designated as the lectotype by Ledoux and Roux (1992) is from “Mikuni-toge” (the Mikuni Pass on the border between Niigata and Nagano prefectures), which is far from “Niohozan” (Fig. 1). Thus, the designated type locality differs between the original description and the lectotype designation. Comparative male genital morphology and macroscale distributions show that populations from “Niohozan” and “Mikuni-toge” should be regarded as the same taxon. Here, the taxon *N. r. niohozana* is upgraded from a subspecies of the species *N. reflexa* to a distinct species, based on marked differences in endophallus morphology and confirmed and putative sympatry with other related species. The confirmed distribution of *N. niohozana* is wide, ranging from Mount Chôkai in the north to Mount Hakusan in the west. The species may be collected with other, related species in areas near Mount Gassan, and Hakusan and the Asahi and Iide Mountains. *Nebria niohozana* can be distinguished from similar species by a larger body size, smaller PPW/EL, and endophallus shape (in particular, Y-shaped lateroapical lobes). The morphological features of the male lectotype (Fig. 3) well match those of the specimens treated here as *N. niohozana* with respect to all macromorphological features, body length (10.18 mm), and PPW/EL (0.296).

**Figures 22–26.**
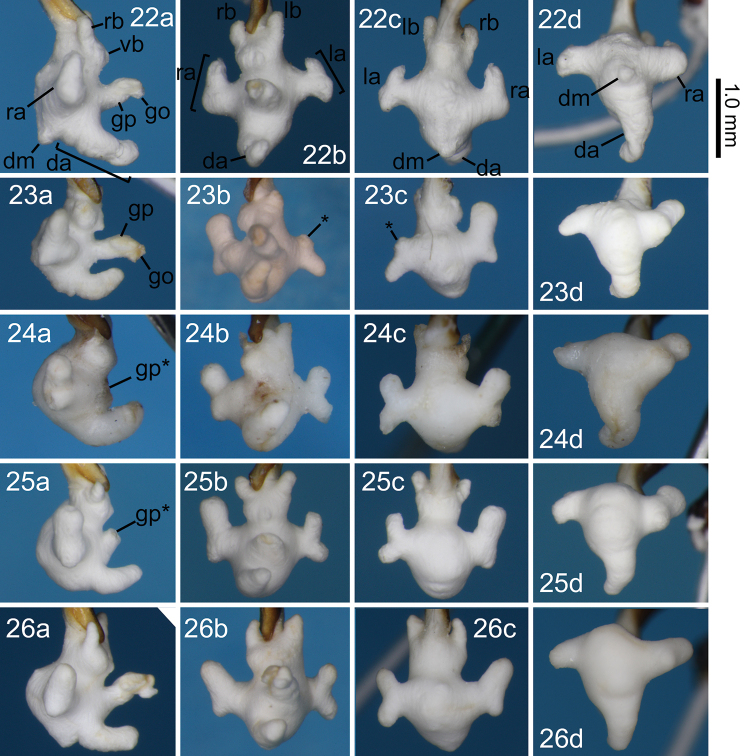
Right lateral (**a**), ventral (**b**), dorsal (**c**), and anterodorsal (**d**) views of the endophallus of *Nebria niohozana* males from Mount Chôkai (**22**), Yumoto, a locality close to the collection site of cotypes in the original description (**23**), Doai, a locality close to the type locality designated in the lectotype designation (**24**), Renge-Onsen (**25**), and Mount Hakusan (**26**). Abbreviations are described in the caption of Figures 17–21.

#### Description.

Body length: ♂, 9.45–11.30 mm (mean ± SD: 10.49 ± 0.39 mm, *n* = 37); ♀, 11.04–12.19 mm (mean ± SD: 11.44 ± 0.36 mm, *n* = 16). PPW/EL: ♂, 0.281–0.334 (mean ± SD: 0.303 ± 0.013, *n* = 37); ♀, 0.273–0.317 (mean ± SD: 0.296 ± 0.014, *n* = 16). Ventral surface of aedeagal apex not concave. Dorsobasal lobe absent. Dorsomedian lobe small or rudimentary, or sometimes absent. Dorsoapical lobe cylindrical, with the apex in an almost ventral direction; slightly curved anteriorly from a lateral view and left-laterally from a posterior view. Right laterobasal lobe small, with the width from a ventral view narrower than the width of the gonopore protrusion from a lateral view. Left laterobasal lobe small, with the width from a ventral view narrower than the width of the gonopore protrusion from a lateral view. Right lateroapical lobe bifurcated at the middle in a Y shape, with one apex directed posterolaterally and the other directed anterolaterally; the posterolateral apex smaller than the anterolateral apex; the anterolateral apex larger than right laterobasal lobe. Left lateroapical lobe bifurcated at the middle in a Y shape, with one apex directed posterolaterally and the other directed anterolaterally; the posterolateral apex similar in size to right laterobasal lobe. Ventrobasal surface with a pair of swelling adjoining each laterobasal lobe; swellings unconnected; ventrobasal swelling small, shaped as a gently sloped mountain from a lateral view.

#### Materials examined.

1♂ (NARO), Fushiogamidake, Mount Chôkai, Yuza-machi, Yamagata Prefecture, Japan, 23.vii.1970, K. Shirahata leg.; 1♀ (NARO), Mount Chôkai, alt. 1700 m, Yuza-machi, Yamagata Prefecture, 20.viii.1957, K. Shirahata leg.; 2♂ (NARO), Mount Gassan, Yamagata Prefecture, Japan, 20.viii.1959, K. Shirahata leg.; 2♂ (NARO), Mount Gando, Yamagata Prefecture, Japan, 6.vi.1954, S. Kimata leg.; 1♀ (KS), Ichimaiishi-sawa, alt. 1375 m, Mount Zaô, Shichikashuku-machi, Miyagi Prefecture, Japan (38.117581N, 140.425892E), 11.ix.2004, K. Sasakawa leg.; 1♂2♀ (NARO), Ôtori-ike, alt. 1000 m, the Asahi Mountains, Yamagata Prefecture, Japan, 23.vii.1959, K. Baba leg.; 1♂ (NARO), Mount Kitamata, the Iide Mountains, Niigata Prefecture, Japan, 15.viii.1964, K. Baba leg.; 1♂ (NARO), Mount Monnai, alt. 1800 m, the Iide Mountains, Niigata Prefecture, 28.vii.1957, K. Baba leg.; 1♂1♀ (NARO), Mount Futastumine, the Iide Mountains, Niigata Prefecture, Japan, K. Baba leg. (1♂, alt. 1600 m, 27.viii.1957; 1♀, 14.vii.1960); 6♂3♀ (KS) , Kuratani-sawa, alt. 480 m, Ôaza-Iine, Okugawa, Nishiaizu-machi, Fukushima Prefecture, Japan, 22–24.ix.2016, H. Itô leg.; 1♂ (NARO), Mount Ôtaki, Aizumisato-machi, Fukushima Prefecture, 15.vii.1950, Y. Kurosawa leg.; 1♂2♀ (KS), Makukawa-Onsen, Tsuchuyuonsen-machi, Fukushima-shi, Fukushima Prefecture, Japan, 23–24.v.2003, K. Sasakawa leg.; 1♂ (NARO), “Egawamura” [a part of Shimogou-machi], Fukushima Prefecture, Japan, 2.vi.1951, Y. Kurosawa leg.; 2♂, Mount Ôjôgo, the Asahi Mountains, Niigata Prefecture, 21.vii.1959, K. Baba leg.; 1♂ (NARO), Sandogoya-Onsen, Nasushiobara-shi, Tochigi Prefecture, Japan, 22.viii.1963, S. Ueno leg.; 2♂ (NARO), Yumoto, Nikkô-shi, Tochigi Prefecture, Japan, K. Tanaka leg. (1♂, 21.vi.1963; 1♂, 22.vi.1963); 2♂, Oze (NARO), on the border between Hinoemata-mura, Fukushima Prefecture and Katashina-mura, Gunma Prefecture, 24.vii.1954, A. Habu leg.; 1♂1♀ (NARO), Shimizu Pass, alt. 1450 m, on the border between Uonuma-shi, Niigata Prefecture and Minakami-machi, Gunma Prefecture, 1.x.1969, K. Baba leg.; 1♂ (NARO), Doai, Minakami-machi, Gunma Prefecture, 2.x.1942, T. Takei leg.; 1♂ (NARO), Mount Tanigawa, Minakami-machi, Gunma Prefecture, 24.x.1955, K. Baba leg.; 4♂3♀ (KS), Mount Naeba, Sakae-mura, Nagano Prefercture, Japan (4♂2♀, 30.vi.2003, W. Toki leg.; 1♀, 3.viii.2003, J. Ogawa leg); 2♀ (NARO), Mount Korenge, Itoigawa-shi, Niigata Prefecture, Japan, 25.vii.1961, K. Baba leg (1♀, no data for altitude; 1♀, alt. 2500 m).; 1♂ (NARO), Renge-Onsen, Mount Shirouma, Niigata Prefecture, Japan, 24.vii.1961, K. Baba leg.; 3♂, Mount Hakusan (NARO), 1300–2000 m, Gifu Prefecture, Japan, 20.vi.1972, K. Tanaka & H. Ohira leg.; 1♂ (NARO), Keimatsudaira, Mount Hakusan, Ishikawa Prefecture, Japan, 1.viii.1961, A. Uchimura leg.

### Nebria (Falcinebria) furcata sp. nov.

Taxon classificationAnimaliaColeopteraCarabidae

9F70BC28-A2A0-5445-A02E-E85A53A74C92

http://zoobank.org/34A51377-A57D-4266-91F0-0AE9D158D797

**Figures 27–30.**
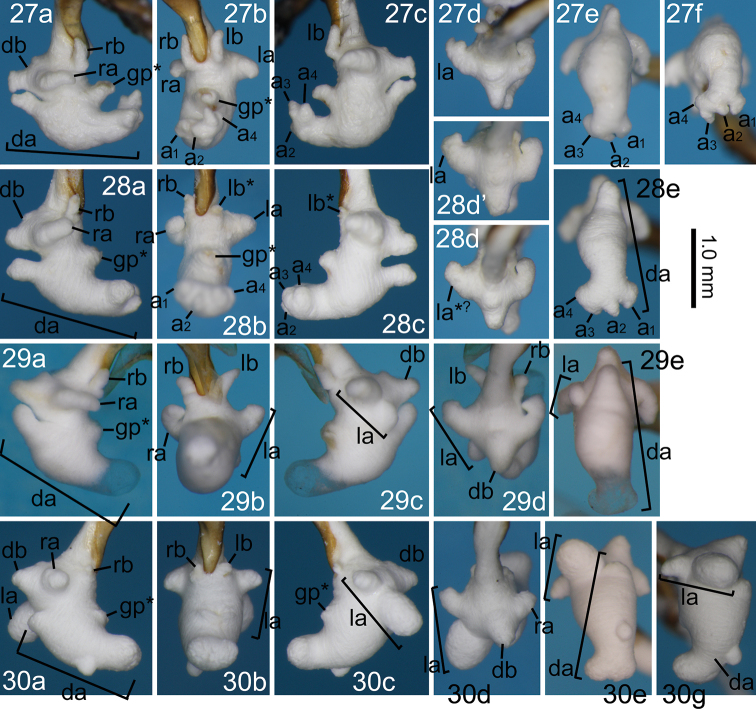
Right lateral (**a**), ventral (**b**), left lateral (**c**), and anterior (**d**) views of the endophallus and dorsal (**e**), posterodorsal (**f**), and left dorsolateral (**g**) views of the dorsoapical lobe of *Nebria* spp. **27***N. furcata* sp. nov., holotype male **28***N. furcata* sp. nov., paratype males from Amô Pass-Shoyashiki **29***N. pisciformis* sp. nov., holotype male **30***N. kuragadakensis* sp. nov., holotype male. db, dorsobasal lobe; other abbreviations are given in Figures 17–21. In *N. furcata* (**27, 28**), a_1-4_ denotes apices of the dorsoapical lobe, and **28d**’ is presented to show individual variation in the focal endophallus structures.

Figs 11 , 27 , 28 Japanese name: Inoue-hime-marukubi-gomimushi

Nebria reflexa : Uéno (1985): 56 (part); Nakane (1963b): 19 (part).

#### Notes.

This species in known from two localities near Mount Hakusan where *N. niohozana* and *N. pisciformis* sp. nov. co-occur. This species is distinguished from *N. niohozana* by a smaller body size and larger PPW/EL (values for male *N. niohozana* from Mount Hakusan: body length, 10.24–10.90 mm; PPW/EL, 0.289–0.321 (*n* = 4); values for females are not shown due to unavailability of specimens), and from *N. pisciformis*, by larger PPW/EL and a trifurcate apical margin of the dorsoapical lobe.

#### Description.

Body length: ♂, 9.27–9.65 mm (mean ± SD: 9.48 ± 0.19 mm, *n* = 3); ♀, 10.21–10.60 mm (mean ± SD: 10.46 ± 0.13 mm, *n* = 4). PPW/EL: ♂, 0.323–0.341 (mean ± SD: 0.334 ± 0.009, *n* = 3); ♀, 0.311–0.320 (mean ± SD: 0.316 ± 0.004, *n* = 4). Ventral surface of aedeagal apex not concave. Dorsobasal lobe present. Dorsomedian lobe absent. Dorsoapical lobe with the basal part protruding dorsally; the protrusion as long as and 1.5 times as wide as the right laterobasal lobe; the apical portion directed ventrally, twice as wide as the subapical constriction; the apical margin divided into four projections. Right laterobasal lobe small, with the width from the ventral view narrower than the width of the gonopore protrusion from the lateral view. Left laterobasal lobe small, with the width from the ventral view narrower than the width of the gonopore protrusion from the lateral view. Right lateroapical lobe small, strongly bent at the middle in an anterior direction. Left lateroapical lobe moderate in size, more or less bifurcate. Ventrobasal surface almost flat, without swellings.

#### Type materials.

Holotype: ♂ (KS), Arashiguchi, Kamiuchinami, Ôno-shi, Fukui Prefecture, Japan, 26.v.2019, S. Inoue leg. Paratypes: 1♀ (KS), same data as the holotype; 2♂3♀ (NARO), Amô Pass–Shoyashiki, Hida-shi, Gifu Prefecture, Japan, 19.vi.1972, K. Tanaka & H. Ohira leg.

#### Etymology.

The specific name derives from the Latin word *furcata* (forked) and refers to the apical margin of the dorsoapical lobe.

### Nebria (Falcinebria) pisciformis sp. nov.

Taxon classificationAnimaliaColeopteraCarabidae

4006A6FE-4332-5A55-94A6-DC99D66B5051

http://zoobank.org/DBFF170F-058B-45D2-BB86-6FC3F261FEF1

Figs 12 , 29 Japanese name: Hakusan-hime-marukubi-gomimushi

Nebria reflexa : Uéno (1985): 56 (part); Nakane (1963b): 19 (part).

#### Notes.

This species is known only from the type locality, Ôshirakawa-dani, which is situated on the eastern foot of Mount Hakusan. It is distinguished from *N. niohozana*, which is known from high altitude areas of Mount Hakusan, by a smaller body size, and from *N. furcata* sp. nov. by a lower PPW/EL and the shape of the dorsoapical lobe.

#### Description.

Body length: ♂, 9.51–9.54 mm (*n* = 2). PPW/EL: ♂, 0.309–0.320 (*n* = 2). Ventral surface of aedeagal apex not concave. Dorsobasal lobe present. Dorsomedian lobe absent. Dorsoapical lobe with the basal part protruding anterodorsally; the protrusion as long as and 1.5 times as wide as right laterobasal lobe; the apical portion directed ventrally, less than twice the width of subapical constriction; the apical margin simple, not furcate. Right laterobasal lobe small, with the width from the ventral view narrower than the width of the gonopore protrusion from the lateral view. Left laterobasal lobe small, with the width from the ventral view narrower than the width of the gonopore protrusion from the lateral view. Right lateroapical lobe small, strongly bent at the middle in an anterior direction. Left lateroapical lobe with the posterior part moderate in size and weakly bent at the middle in an anterolateral direction; the anterior part weakly swollen. Ventrobasal surface almost flat, without swelling.

#### Type materials.

Holotype: ♂ (NARO), Ôshirakawa-dani, 800–1250 m, Shirakawa-mura, Gifu Prefecture, Japan, 19.vi.1972, K. Tanaka & H. Ohira leg.; 1♂, same data as the holotype.

#### Etymology.

The specific name derives from a combination of the Latin words *piscis* (fish) and -*formis* (-shaped) and refers to the dorsal view of the dorsoapical lobe.

### Nebria (Falcinebria) kuragadakensis sp. nov.

Taxon classificationAnimaliaColeopteraCarabidae

ADD75902-0522-5474-923A-0A0A374A7A05

http://zoobank.org/C0496598-8A20-49F9-88FE-1CE7F6DCE9A4

Figs 13 , 30 Japanese name: Kuragadake-hime-marukubi-gomimushi

Nebria reflexa : Uéno (1985): 56 (part); Nakane (1963b): 19 (part).

#### Notes.

This species is known only from the type locality, Mount Kuragatake, a low mountain located northwest of Mount Hakusan. It is distinguished from *N. niohozana*, which is also distributed on Mount Hakusan, by a larger PPW/EL.

#### Description.

Body length: ♂, 9.59 mm (*n* = 1); ♀, 10.28 mm (*n* = 1). PPW/EL: ♂, 0.342 (*n* = 1); ♀, 0.334 (*n* = 1). Ventral surface of aedeagal apex not concave. Dorsobasal lobe present. Dorsomedian lobe absent. Dorsoapical lobe with the basal part weakly swollen; the left dorsolateral surface behind the subapical constriction weakly swollen; the apical portion directed ventrally, less than twice the width of the subapical constriction; the apical margin very slightly bifurcated. Right laterobasal lobe small, with the width from the ventral view narrower than the width of the gonopore protrusion from the lateral view. Left laterobasal lobe small, with the width from the ventral view narrower than the width of the gonopore protrusion from the lateral view. Right lateroapical lobe small, weakly bent at the middle in an anterolateral direction. Left lateroapical lobe with the posterior part small and weakly bent at the middle in an anterolateral direction; the anterior part largely swollen, directed left-dorsoposteriorly. Ventrobasal surface almost flat, without swelling.

#### Type materials.

Holotype: ♂ (NARO), Mount Kuragadake, Kanazawa-shi, Ishikawa Prefecture, Japan, 20.v.1962, S. Takaba leg.; Paratype: 1♀ (NARO), same data as the holotype.

#### Etymology.

The specific name refers to Mount Kuragadake, the type locality of this new species.

### Nebria (Falcinebria) dichotoma sp. nov.

Taxon classificationAnimaliaColeopteraCarabidae

990F6C74-789A-5924-9E14-81892D4AF78A

http://zoobank.org/A6290AFF-8569-4594-BFDC-BEEF9C11B1DF

**Figures 31–35.**
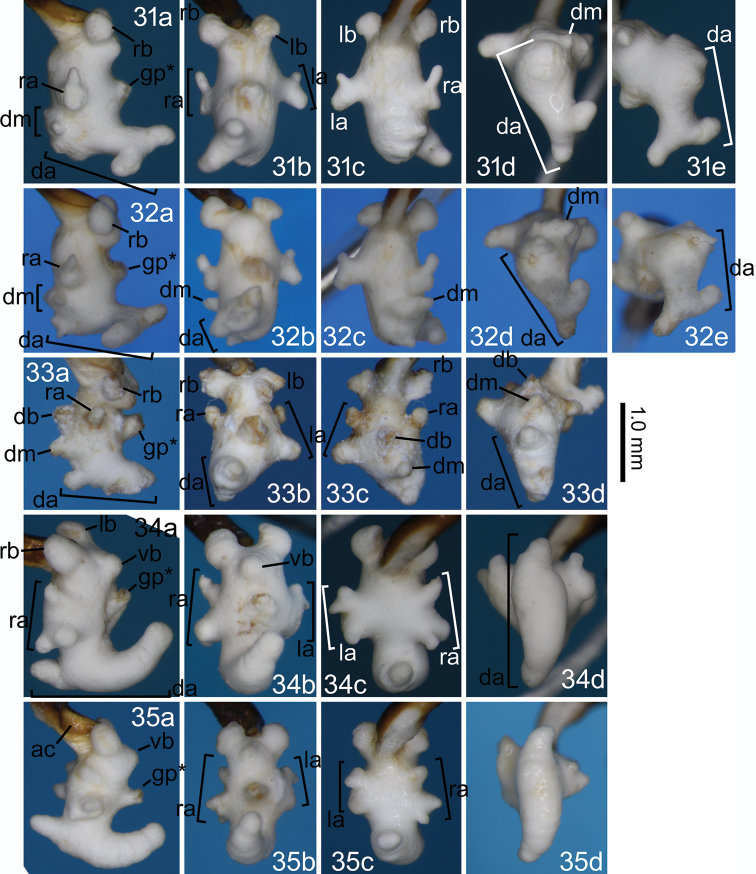
Right lateral (**a**), ventral (**b**), dorsal (**c**), and posterodorsal (**d**) views of the endophallus and left laterodorsal view of the dorsoapical lobe (**e**) of *Nebria* spp. **31***N. dichotoma* sp. nov., holotype male **32***N. dichotoma* sp. nov., paratype male from the type locality **33***N. uenoi*, male from the type locality **34***N. chugokuensis* sp. nov., holotype male **35***N. chugokuensis*, paratype male from Sandan-kyô. ac, concavity of aedeagal apex; remaining abbreviations are provided in Figures 17–21.

Figs 14 , 31 , 32 Japanese name: Dando-hime-marukubi-gomimushi

Nebria reflexa : Uéno (1985): 56 (part); Nakane (1963b): 19 (part).

#### Notes.

This species is known only from the type locality, Mount Takanosu. Among known related species, it is similar to *N. chugokuensis* sp. nov. in having a relatively large-sized body and larger PPW/EL but is distinguished by a lack of concavity on the ventral surface of the aedeagal apex.

#### Description.

Body length: ♂, 9.80–10.34 mm (mean ± SD: 10.14 ± 0.22 mm, *n* = 5); ♀, 10.49–11.12 mm (mean ± SD: 10.79 ± 0.30 mm, *n* = 6). PPW/EL: ♂, 0.314–0.335 (mean ± SD: 0.322 ± 0.010, *n* = 5); ♀, 0.302–0.324 (mean ± SD: 0.312 ± 0.008, *n* = 6). Ventral surface of aedeagal apex not concave. Dorsobasal lobe absent. Dorsomedian lobe small, directed right-laterally. Dorsoapical lobe with two small protrusions at the basal part; the apical portion directed ventrally, bifurcated in a Y-shape. Right laterobasal lobe large, with the width from a ventral view wider than the width of the gonopore protrusion from a lateral view. Left laterobasal lobe large, with the width from a ventral view wider than the width of the gonopore protrusion from a lateral view. Right lateroapical lobe bifurcated at the base in a V shape, with one apex directed posterolaterally and the other directed anterolaterally; the posterolateral apex very small; the anterolateral apex smaller than right laterobasal lobe. Left lateroapical lobe bifurcated at the middle in a Y shape, with one apex directed posterolaterally and the other directed anterolaterally; the posterolateral apex smaller than the anterolateral apex; the anterolateral apex smaller than the right laterobasal lobe. Ventrobasal surface with a pair of swelling adjoining each laterobasal lobe; swellings conjoined; ventrobasal swelling absent.

#### Type materials.

Holotype: ♂ (NARO), Mount Takanosu (= Mount Dando), alt. 1000 m, Shitara-machi, Aichi Prefecture, Japan, 8.vi.1971, K. Tanaka & H. Ohira leg. Paratypes (NARO): 2♂4♀, same data as the holotype; 2♂2♀, same locality and collector (1♂2♀, 24.vi.1972; 1♂, 4.viii.1972).

#### Etymology.

The specific name derives from the Greek word *dichotoma* (divided into two) and refers to the apical portion of the dorsoapical lobe.

### Nebria (Falcinebria) uenoi

Taxon classificationAnimaliaColeopteraCarabidae

Nakane, 1963

B7617C5A-20BD-5A82-AEF0-3927D51C104C

Figs 15 , 33 Japanese name: Kinki-hime-marukubi-gomimushi

Nebria reflexa uenoi : Nakane (1963a): 218 (original description), type locality: “Kasuga, Nara, Honshu”; Nakane (1963b): 19, plate 10 fig. 1b.
Nebria reflexa : Uéno (1985): 56 (part).
Nebria (Orientonebria) reflexa uenoi : Farkač and Janata (2003): 94.
Nebria (Falcinebria) reflexa uenoi : Ledoux and Roux (2005): 831, plate-fig. 627; Yoshitake et al. (2011): 32; Huber (2017): 50; Yoshimatsu et al. (2018): 37.
Nebria (Falcinebria) reflexa ssp.: Yoshitake et al. (2011): 33.

#### Notes.

This species is known only from the type locality, Mount Kasuga. Although similar in body size and PPW/EL to *N. furcata*, *N. shirokawa*, and *N. kuagadakensis*, it is distinguished from these species by the shape of the endophallus.

#### Description.

Body length: ♂, 9.92–10.00 mm (*n* = 2); ♀, 9.94–10.56 mm (mean ± SD: mean ± SD: 10.34 ± 0.34 mm, *n* = 3). PPW/EL: ♂, 0.332–0.345 (*n* = 2); ♀, 0.314–0.330 (mean ± SD: 0.324 ± 0.009, *n* = 3). Ventral surface of aedeagal apex not concave. Dorsobasal lobe present. Dorsomedian lobe small. Dorsoapical lobe with basal part protruding posterodorsally; protrusion smaller than the right laterobasal lobe; the apical portion directed ventrally. Right laterobasal lobe large, with the width from the ventral view wider than the width of the gonopore protrusion from the lateral view. Left laterobasal lobe large, with the width from the ventral view wider than the width of the gonopore protrusion from the lateral view. Right lateroapical lobe small, simply swollen. Left lateroapical lobe bifurcated at the base, with one apex directed posterolaterally and the other directed anterolaterally; the posterolateral apex larger than the anterolateral apex; the posterolateral apex larger than the anterolateral apex and slightly smaller than the right laterobasal lobe. Ventrobasal surface with a pair of swelling adjoining each laterobasal lobe; swellings unconnected; ventrobasal swelling absent.

#### Materials examined.

2♂3♀ (NARO), Mount Kasuga, Nara Prefecture, Japan (1♀, 17.v.1952, H. Ishida leg.; 2♂, 27.v.1950, S. Ueno leg.; 2♀, 29.v.1950, S. Ueno leg.)

### Nebria (Falcinebria) chugokuensis sp. nov.

Taxon classificationAnimaliaColeopteraCarabidae

38914AA3-FF7A-5B01-84F5-A55824A4CF38

http://zoobank.org/70FDF936-491C-4637-B851-35202CE729EA

Figs 16 , 34 , 35 Japanese name: Chûgoku-hime-marukubi-gomimushi

Nebria reflexa : Uéno (1985): 56 (part); Nakane (1963b): 19 (part).
Nebria (Falcinebria) reflexa uenoi : Yoshiake et al. (2011): 33 (part).

#### Notes.

This species is widely distributed in the Chûgoku Mountains. It is readily distinguished from other species previously considered *N. reflexa* distributed in Honshû by the concavity of the ventral surface of the aedeagal apex. It is distinguished from *N. hikosana*, described from nearby northern Kyûshû, by two pairs of setae on the ventral side of the sterna, versus one pair in *N. hikosana*.

#### Description.

Body length: ♂, 9.97–10.32 mm (mean ± SD: 10.12 ± 0.13 mm, *n* = 5); ♀, 10.69–11.75 mm (mean ± SD: 11.22 ± 0.53 mm, *n* = 3). PPW/EL: ♂, 0.312–0.324 (mean ± SD: 0.320 ± 0.005, *n* = 5); ♀, 0.305–0.317 (mean ± SD: 0.313 ± 0.007, *n* = 3). Ventral surface of aedeagal apex deeply concave. Dorsobasal lobe absent. Dorsomedian lobe absent. Dorsoapical lobe with the basal part protruding dorsally; the protrusion as long as and narrower than the right laterobasal lobe; apical portion directed anteroventrally. Right laterobasal lobe large, with the width from a ventral view wider than the width of the gonopore protrusion from a lateral view. Left laterobasal lobe large, with the width from a ventral view wider than the width of the gonopore protrusion from a lateral view. Right lateroapical lobe bifurcated at the middle, with one apex directed posterolaterally and the other directed anterolaterally; the posterolateral apex further bifurcated. Left lateroapical lobe bifurcated at the middle, with one apex directed posterolaterally and the other directed anterolaterally; the anterolateral apex further bifurcated. Ventrobasal surface without swellings adjoining the laterobasal lobes; ventrobasal swelling large, semispherical from a lateral view.

#### Type materials.

Holotype: ♂ (NARO), Mount Azuma, Hiwa-chô, Shôbara-shi, Hiroshima Prefecture, Japan, 27.vii.1976, S. Nakamura leg. Paratypes (NARO): 1♂, same description as the holotype; 2♂3♀, Sandan-kyô, Akiôta-chô, Hiroshima Prefecture, Japan (1♂1♀, 30.v.1957, K. Baba leg.; 1♂1♀, 31.v.1957, K. Baba leg.; 31.v.1970, I. Okamoto leg.); 1♂, Chômon-kyô, Atou, Yamaguchi-shi, Yamaguchi Prefecture, Japan, 18.v.1975, K. Tanaka leg.

#### Etymology.

The specific name refers to the Chûgoku Mountains, where this new species is distributed.

## Discussion

**Figure 36.**
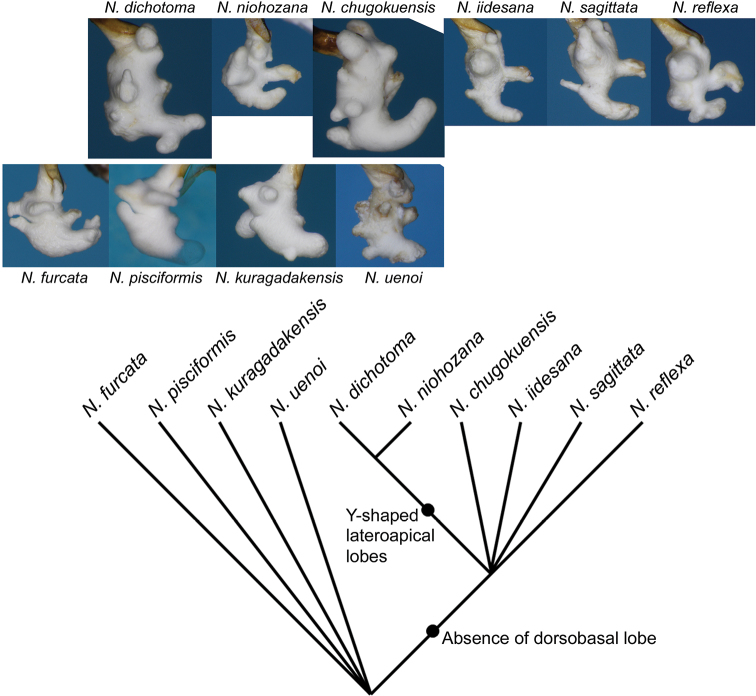
Phylogenetic relationships of the 10 *Nebria* species inferred from two unambiguous synapomorphies. Right lateral views of the endophallus of all species are also shown to demonstrate the morphological diversity.

The previously recognized *N. reflexa* is composed of several “cryptic species” that are distinguished by the shape of the endophallus, an organ of the male genitalia (Fig. 36). Although these species are broadly similar in external appearance, some are distinguishable by morphometric values (Fig. 4). Sympatric occurrence was confirmed for one species pair (*N. iidesana* and *N. niohozana*), providing definitive evidence that these two species are not subspecies of a single species (i.e., geographical races) but rather are reproductively isolated and distinct. Further cryptic species previously considered as *N. reflexa* may remain to be discovered. Some Honshû populations recorded as *N. reflexa* (e.g., Kasahara 1985; Satake and Kasahara 1985; The Japan Coleopterological Society 2007; Morita and Hirai 2010; Yoshitake et al. 2011; Hiramatsu and Usio 2018; Mori 2018; Yoshimatsu et al. 2018; Nakahama et al. 2019) could not be identified here, in some instances due to the unavailability of male specimens (Yoshitake et al. 2011; Yoshimatsu et al. 2018). In addition, populations on Shikoku (Yoshida et al. 2007; Yoshitomi et al. 2012) and those on Kyûshû from localities outside the type locality of *N. r. hikosana* (e.g., Nishida 2005) remain unexamined. Future examination of specimens from these populations is encouraged.

Although this work was not comprehensive of all collection sites, as described above, comparative morphology, mainly of the endophallus, and comparison of distribution patterns provided insight into the differentiation process of these species in the Japanese Archipelago. Among the endophallus characters observed here, the absence of a dorsobasal lobe in some species is noteworthy. In most *Nebria* species for which the endophallus has been examined (e.g., Dubko and Matalin 2002; Ledoux and Roux 2005; Sasakawa 2016; Sasakawa and Kubota 2006) and in a species of *Leistus* (Morita 2012), the sister taxon of *Nebria*, the dorsobasal lobe is well developed, suggesting that the absence of a dorsobasal lobe is an apomorphic character condition in *Nebria*. Thus, the six species lacking a dorsobasal lobe, namely *N. reflexa*, *N. sagittata*, *N. iidesana*, *N. niohozana*, *N. dichotoma*, and *N. chugokuensis*, are considered to form a clade among the species examined. Within this clade, *N. niohozana* is likely the most derived, as it has the largest body size and widest distribution range, both of which are typical characteristics of derived species and/or lineages in other groups of Carabidae (e.g., Ishikawa 1979; Sota et al. 2005; Fujisawa et al. 2019). *Nebria niohozana* is likely sister to *N. dichotoma*, as they share Y-shaped lateroapical lobes. This lobe shape is found only in these two species and is considered to be apomorphic within the genus (Dubko and Matalin 2002; Ledoux and Roux 2005; Sasakawa and Kubota 2006; Sasakawa 2016). Phylogenetic relationships of the 10 species presented here are inferred as shown in Figure 36. Importantly, four species of the basal lineages (*N. furcata*, *N. pisciformis*, *N. kuragadakensis*, and *N. uenoi*) and *N. dichotoma*, which is the putative sister taxon of the derived species *N. niohozana*, are all distributed in the western Chûbu and eastern Kinki regions. This pattern indicates that the initial differentiation of these species likely occurred in these regions. This finding is similar to those in other flightless groups of Carabidae, e.g., the Carabus subgenus Ohomopterus (Carabini), the Nebria subgenus Sadonebria (Nebrini), *Apatrobus* (Patrobini), and *Jujiroa* (Platynini), in which the most ancestral species are distributed in the western Chûbu and/or eastern Kinki region and derived species occur in other regions (Sasakawa 2006; Sasakawa and Kubota 2006; Dejima and Sota 2017; Fujisawa et al. 2019). The western Chûbu and eastern Kinki regions are therefore likely to have been the main areas in Japan where the initial differentiation of various groups of Carabidae occurred.

## Supplementary Material

XML Treatment for Nebria (Falcinebria) reflexa

XML Treatment for Nebria (Falcinebria) sagittata

XML Treatment for Nebria (Falcinebria) iidesana

XML Treatment for Nebria (Falcinebria) niohozana

XML Treatment for Nebria (Falcinebria) furcata

XML Treatment for Nebria (Falcinebria) pisciformis

XML Treatment for Nebria (Falcinebria) kuragadakensis

XML Treatment for Nebria (Falcinebria) dichotoma

XML Treatment for Nebria (Falcinebria) uenoi

XML Treatment for Nebria (Falcinebria) chugokuensis

## Supplementary materials

Supplementary material 1 Table S1. Raw data for discriminant analysis Data type: statistical data https://binary.pensoft.net/file/370835This dataset is made available under the Open Database License (http://opendatacommons.org/licenses/odbl/1.0/). The Open Database License (ODbL) is a license agreement intended to allow users to freely share, modify, and use this Dataset while maintaining this same freedom for others, provided that the original source and author(s) are credited.Kôji Sasakawa
